# Dynamic Thermo-Mechanical Properties of Carbon Nanotube Resin Composite Films

**DOI:** 10.3390/polym16233307

**Published:** 2024-11-27

**Authors:** Ying Wang, Zhouyi Li, Yan Liu, Penghao Pei

**Affiliations:** 1 School of Civil Engineering and Architecture, Xi’an University of Technology, Xi’an 710048, China; 13782963207@163.com (Y.W.); lizhouyi1993@163.com (Z.L.); 2 College of Physics and Optoelectronic Engineering, Shenzhen University, Shenzhen 518060, China; 3 School of Aeronautics, Northwestern Polytechnical University, Xi’an 710072, China; 15529583078@163.com

**Keywords:** carbon nanotube films, epoxy resin, composite material, dynamic thermo-mechanical analysis

## Abstract

In this paper, we prepared carbon nanotube (CNT) epoxy composite films and conducted tensile experiments at various temperatures (−40 °C, −10 °C, 20 °C, and 50 °C) and frequencies (1 Hz, 10 Hz, and 20 Hz) using Dynamic Mechanical Analysis (DMA). This study reveals the effects of temperature and frequency on the mechanical properties of CNT films and CNT epoxy composite films. The results indicate that the energy storage modulus of the pure CNT film is approximately 13 times greater than that of the composite material at 20 °C. Additionally, the loss factor of the composite material is about 25 times that of pure epoxy resin and 7 times that of pure CNT film. These findings suggest that the presence of epoxy resin reduces the elastic deformation capacity of the CNT film while enhancing its damping properties. The mechanical properties of CNT films and CNT epoxy composites at varying temperatures and frequencies investigated in this work offer valuable insights for future applications and studies of CNT films and CNT epoxy composites in diverse environments.

## 1. Introduction

In recent years, nanomaterials have experienced rapid development and widespread application. Compared to traditional materials, nanomaterials possess unique characteristics, including the small size effect, surface effect, quantum size effect, and macroscopic quantum tunneling [1]. Among these, one-dimensional nanomaterials, particularly inorganic nanofiller carbon nanotubes (CNTs), hold significant promise for various applications in fields such as bio-based materials and electronics. Carbon nanotubes are renowned for their exceptional chemical and physical properties, making them valuable in mechanics, electronics, and chemistry [2,3,4,5]. However, due to the unique molecular structure of CNTs, the direct incorporation of CNT powder into resin can lead to agglomeration [6], which adversely affects the material’s properties. CNT thin films, produced using the Floating Catalyst Chemical Vapor Deposition (FCCVD) method, are two-dimensional structures formed by arrays of carbon nanotubes [7]. These films exhibit remarkable cross-scale characteristics, enabling them to demonstrate excellent performance at the macroscopic level. 

To enhance the understanding of the mechanical and physical properties of carbon nanotube (CNT) films and their composites, researchers have undertaken extensive investigations. Tan Wei et al. [8] fabricated CNT films and CNT resin composite films, utilizing uniaxial tensile tests to evaluate the tensile stress–strain responses of both film types. Their results indicated that the modulus and yield strength of CNT resin composite films were significantly improved compared to the corresponding values of CNT films. Xia et al. [9] investigated and developed highly conductive silver-modified carbon nanotube films, demonstrating that these silver-ion-modified films can effectively reduce substrate damage caused by lightning strikes, thereby serving as an efficient protective layer against lightning strikes. Qu Shuxuan et al. [10] developed a self-sensing composite material with online damage-monitoring capabilities, capitalizing on the exceptional electromechanical response characteristics of CNT films to facilitate real-time damage assessment in composite materials. Rogozhkin et al. [11] conducted a study examining the influence of the physical properties of single-walled carbon nanotube (SWCNT) films on the monitoring of thermoset polymers during both their manufacturing and application phases. They specifically isolated and assessed the impact of film thickness on the monitoring properties of these polymers. Sun et al. [12] prepared ternary polymer composites by incorporating acidified carbon nanofibers (CNF) and sulfonated polystyrene-block-poly(ethylene-co-butylene)-block-polystyrene (SSEBS) into epoxy resin through a solution processing method. The synergistic strengthening and toughening effects of the hybrid fillers were investigated using quasi-static mechanical tests and microscopic morphology characterization. The findings indicated that the addition of SSEBS and CNF enhanced the fracture toughness and tensile strength of the pure epoxy resin. Li et al. [13] explored the low-temperature mechanical properties, gas barrier characteristics, and thermal properties of modified resin by preparing multi-walled carbon nanotube (MWCNT)-modified epoxy resin. Their results demonstrated that, in comparison to the epoxy matrix, the gas permeability coefficient of the nanocomposites containing MWCNTs was reduced, while the tensile strength and destructive strain at low temperatures (77 K) increased. Additionally, the thermal stability and glass transition temperature of the modified resins were improved.

Due to their remarkable mechanical and physical properties, carbon nanotubes (CNTs) are commonly utilized to reinforce phase materials, thereby enhancing the mechanical characteristics of the original matrix. Wu Mengya et al. [14] employed acoustic emission technology to investigate the effects of curing temperature and the incorporation of carbon nanotubes on the acoustic emission signals of epoxy resin. Their results indicated that, as the concentration of carbon nanotubes increased, the amplitude of the acoustic emission signals recorded by the acoustic emission instrument gradually decreased, concurrently improving the strength and elongation at break of the epoxy resin. Kordzangeneh et al. [15] examined the influence of multiwalled carbon nanotube (MWCNT) reinforcement in epoxy resin at varying concentrations on the creep response and residual tensile properties of the nanocomposites following creep. Their findings revealed that the residual strength and stiffness of all samples diminished after partial creep tests compared to pre-creep values; however, the reduction in residual strength and stiffness for the composites was less pronounced than that observed in pure epoxy resin. To elucidate the discrepancies in mechanical enhancement provided by CNTs across different matrices, Zhu Xiufang et al. [16] conducted quasi-static tensile tests, three-point bending tests, and dynamic impact experiments on two types of composites: CNT-reinforced resin-based and aluminum alloy matrices, thereby clarifying the reinforcement mechanisms of CNTs in various matrices. Liu Haoran et al. [17] utilized a light air cannon to perform high-speed impact tests on CNT films with varying resin contents, revealing the performance of CNT resin composite films against transverse high-speed impacts and the influence of resin content on impact performance. Xiao et al. [18] investigated the impact resistance of CNT films, both with and without cross-linking, through micro ballistic impact experiments and coarse-grained molecular dynamics (CGMD) simulations. Their research demonstrated that the introduction of cross-linked structures enhances the interaction between adjacent CNTs by increasing covalent bonds, thereby significantly improving the energy dissipation efficiency of CNT films. Hasanin et al. [19] successfully formulated a multifunctional composite material by fabricating glass fiber-epoxy resin sheets in the presence of carbon nanotubes (CNTs) and confirmed that the incorporation of CNTs not only enhanced the mechanical properties of the composites but also imparted self-healing capabilities, particularly in their ability to withstand bullet impacts, further validating the effectiveness of CNT integration in improving the resilience of composites. The role of CNTs in enhancing the toughness and durability of composites was thus affirmed. Wang et al. [20] conducted a comparative analysis of the friction properties of primitive polylactic acid (PLA) nanofibers and enzymatically degraded polylactic acid (ED-PLA) nanofibers under various conditions. Their findings indicated that the reciprocating mode more effectively demonstrates the results of the friction tests, providing valuable technical guidance for friction tests on fiber membrane materials.

While existing research offers a substantial experimental and theoretical foundation for understanding the mechanical properties of carbon nanotubes (CNTs) and their composites under quasi-static conditions, the mechanical behavior of these materials under dynamic conditions remains inadequately explored [21]. Most current studies have primarily focused on the influence of carbon nanotubes on the properties of epoxy resin matrices, highlighting that the incorporation of carbon nanotubes enhances the damping characteristics of these resins [22,23]. For instance, Pan Shengqi et al. [24] proposed an improved Biot model to simulate the frequency-dependent damping characteristics of CNT-reinforced epoxy resin nanocomposites. Their findings indicated that, at a CNT content of 0.4 wt %, the first-order damping ratio of the composites reached a maximum of 0.591, which represents a 41% increase compared to pure epoxy resin. Consequently, this paper employs dynamic mechanical analysis (DMA) to conduct tensile tests on CNT films and CNT-epoxy resin composites. The objective is to elucidate the effects of temperature and frequency on the mechanical properties of both CNT films and CNT-epoxy resin composites, while also comparing their mechanical properties across varying temperatures and frequencies. This research aims to provide valuable insights and guidance for the future application and investigation of CNT films and CNT-epoxy resin composites in diverse environments.

## 2. Material Preparation and Test Methods

### 2.1. Experimental Materials

The fabrication of carbon nanotube-reinforced polymer matrix composites necessitates the integration of carbon nanotube (CNT) powder into the polymer matrix, a process that poses challenges such as agglomeration and uneven dispersion of the carbon nanotubes [6]. In contrast, CNT thin films produced through the floating catalytic chemical vapor deposition (FCCVD) method exhibit a network structure characterized by the intertwining and entanglement of CNTs, which facilitates improved resin infiltration during the composite preparation process. FCCVD is a continuous production method for carbon nanotube fibers, employing liquid carbon-containing organic compounds, such as ethanol or acetone, as carbon sources, along with organic salts containing iron as catalysts, and sulfur-containing organic compounds as additives. Upon injection into the furnace via a liquid injection pump, the organic compounds, including the carbon source and catalyst, volatilize into a gaseous state at elevated temperatures. With the assistance and catalysis of sulfur and iron, carbon nanotubes are synthesized from the carbon source, resulting in the self-assembly of numerous carbon nanotubes into smoke-like aerogels. Carbon nanotube films can be obtained by continuously depositing the carbon nanotube aerogels onto collection rollers as the carrier gas exits the furnace tubes [25].

**Figure 1:**
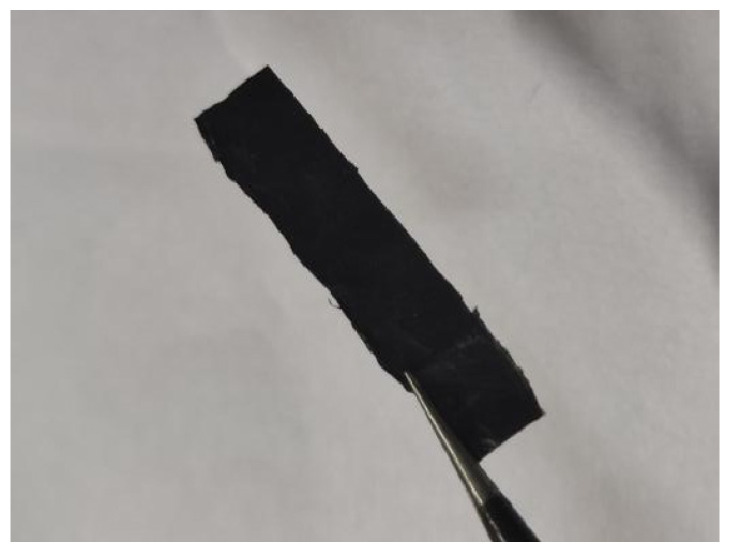
CNT film specimen.

The carbon nanotube (CNT) films were prepared by multiwalled carbon nanotubes (MWCNTs) through the floating catalytic method developed by Chengdu Jiacai Technology Co., Ltd. (Chengdu, China). The resultant films exhibited a thickness of approximately 20 μm, with the CNTs uniformly distributed throughout the matrix. Specimens measuring 30 mm in length and 7 mm in width were subsequently cut from these films, as illustrated in Figure 1. CNT-epoxy resin composites were produced by infiltrating the CNT films with a mixed solution of epoxy resin and acetone. The epoxy resin was combined with a curing agent in a mass ratio of 10:3, followed by dilution with acetone to achieve various mass concentrations of the mixed solution. The epoxy resin utilized in this study was bisphenol-A IN2, which primarily consists of bisphenol-A-epichlorohydrin, 4,4′-isopropylidenediphenol diglycidyl ether, and oligomeric reaction products of formaldehyde with 1-chloro-2,3-epoxypropane and phenol. This resin is thermosetting and can undergo polymerization with a variety of curing agents, catalysts, and additives. The principal component of the curing agent is 1,3-cyclohexanedimethanamine. During the curing process, the amine in the curing agent reacts with the epoxy groups, resulting in the opening of the epoxy rings to form ring-opening intermediates. These intermediates subsequently react with additional epoxy groups, leading to the formation of a cross-linked network structure.

**Figure 2:**
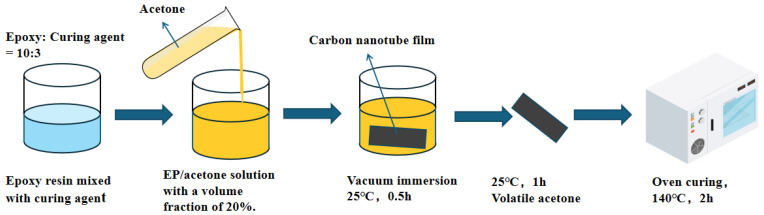
Schematic diagram of CNT epoxy resin film sample preparation.

**Figure 3:**
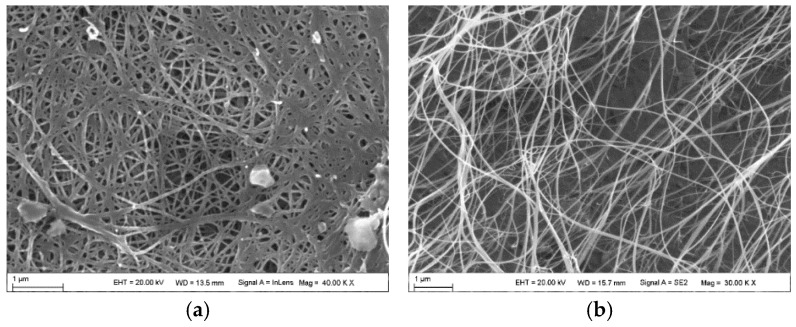
Microscopic morphology characterization of CNT film and CNT epoxy resin film. (**a**) Pure CNT film (**b**) CNT epoxy resin film.

To investigate the impact of resin on the mechanical properties of carbon nanotube (CNT) films, the experimental procedure was conducted as outlined in the preceding steps, utilizing a mixed solution with a mass fraction of 20%. The CNT film was thoroughly impregnated in the prepared resin solution and subjected to a vacuum for a duration of thirty minutes. Subsequently, the film was removed and allowed to rest at room temperature for one hour to facilitate the volatilization of acetone. Following this, the film was placed in an oven maintained at a constant temperature of 140 °C for a curing period of two hours, resulting in the formation of the CNT epoxy resin composite material. The sample preparation process is illustrated in Figure 2, while the microscopic morphology of the CNT film is characterized in Figure 3.

### 2.2. Experimental Methods

**Figure 4:**
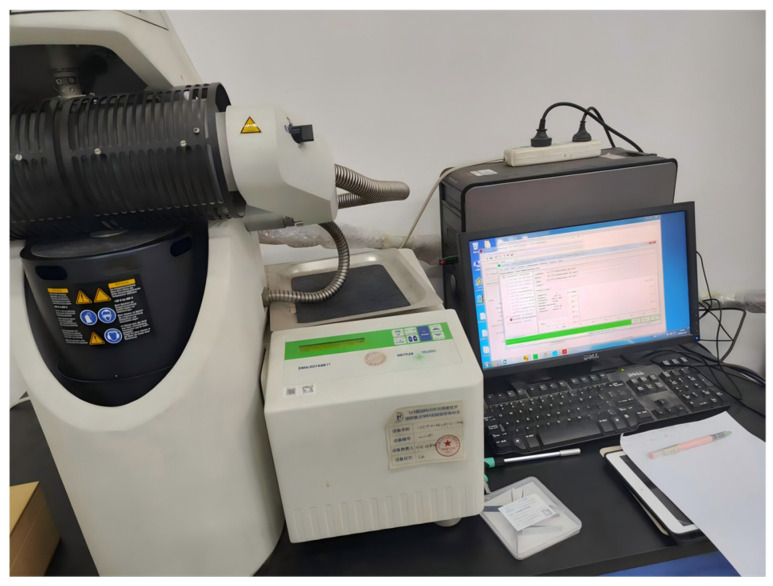
The dynamic thermo-mechanical analysis test machine.

**Figure 5:**
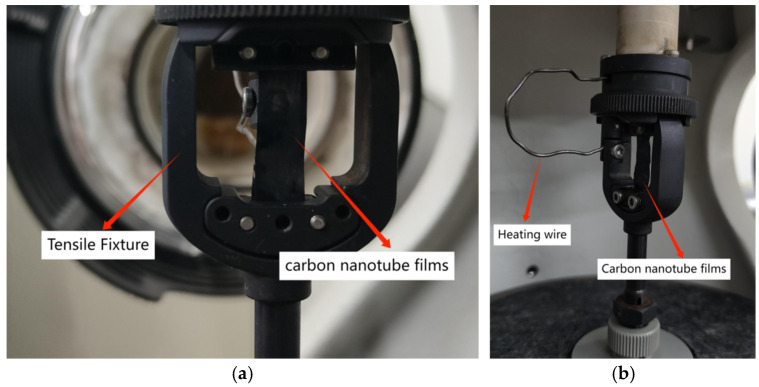
The clamp and the specimen: (**a**) Physical image of Tensile test clamp; (**b**) Physical diagram of the relationship between the heating wire and the specimen position.

In this study, a dynamic thermo-mechanical analyzer (DMA) was utilized to perform dynamic mechanical experiments at various temperatures and frequencies, with the objective of assessing the mechanical properties of materials in relation to these parameters. The schematic representation of the testing apparatus is illustrated in Figure 4. The DMA was employed to apply tensile loads to pure carbon nanotube (CNT) films and CNT epoxy resin films at ambient temperatures of −40 °C, −10 °C, 20 °C, and 50 °C, corresponding to frequencies of 1 Hz, 10 Hz, and 20 Hz, in order to determine the associated mechanical properties. The physical representation of the fixture specimen is shown in Figure 5, and a CNT resin composite film measuring 30 mm × 7 mm was affixed to the fixture as the specimen.

## 3. Results and Discussion

### 3.1. Effect of Temperature on Material Properties

**Figure 6:**
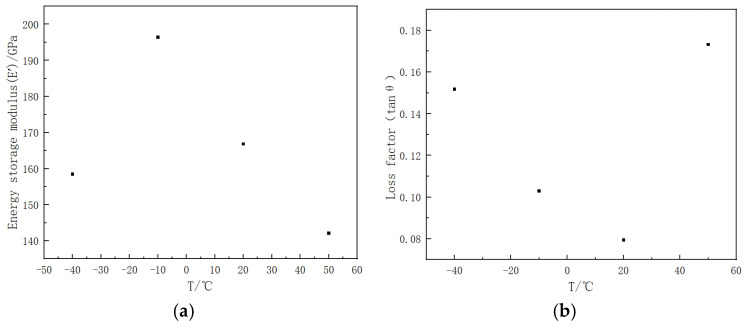
Different mechanical properties of CNT film under different temperatures (−40 °C, −10 °C, 20 °C, and 50 °C): (**a**) energy storage modulus, (**b**) loss factor.

**Figure 7:**
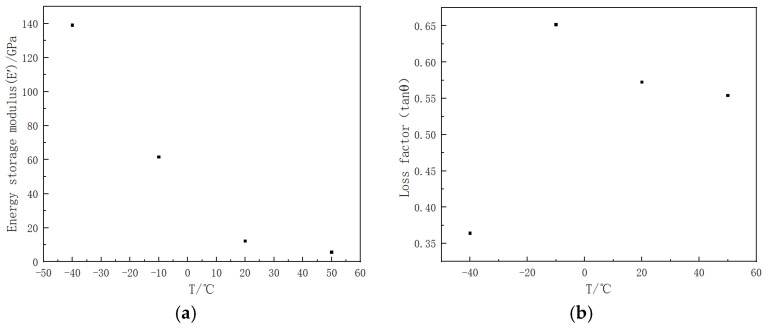
Different mechanical properties of CNT epoxy resin film under different temperatures (−40 °C, −10 °C, 20 °C, and 50 °C): (**a**) energy storage modulus, (**b**) loss factor.

The tensile mechanical properties of pure carbon nanotube (CNT) films and CNT epoxy resin films, obtained through experimental methods, are presented in Figure 6 and Figure 7. These figures illustrate the energy storage modulus and loss factor of the materials at ambient temperatures of −40 °C, −10 °C, 20 °C, and 50 °C, respectively.

Figure 6 presents the energy storage modulus and loss factor CNT film at a frequency of 1 Hz across a range of temperatures. It is noteworthy that the energy storage modulus of the pure CNT film attains its maximum value of approximately 196 GPa at −10 °C. Following this peak, an increase in temperature results in a decrease in the energy storage modulus by approximately 15%. Furthermore, the loss factor of the material demonstrates a gradual reduction as ambient temperatures rise, reaching a minimum value of 0.08 at −40 °C, −10 °C, and 20 °C. At an ambient temperature of 50 °C, the loss factor of the pure CNT film increases to approximately 0.17, which is twice the value observed at 20 °C.

When the selected frequency is set to 1 Hz, the energy storage modulus and loss factor of CNT epoxy resin films at various temperatures are depicted in Figure 7. The figure illustrates that, for the CNT epoxy resin films, an increase in temperature results in a significant decrease in the energy storage modulus. Specifically, at −10 °C, the energy storage modulus is approximately 56% lower than that observed at −40 °C. Furthermore, when the material is exposed to temperatures of −40 °C and −10 °C, the loss factor increases from 0.36 to a maximum of 0.65 as the ambient temperature rises, indicating enhanced damping performance. In contrast, at temperatures of −10 °C, 20 °C, and 50 °C, the loss factor gradually decreases with increasing ambient temperature; however, the extent of this reduction is relatively modest, ranging from approximately 3% to 12%.

Because the deformation energy that can be stored by chain segments in a free-motion state is lower than that stored in a frozen or bound state [26], as the temperature gradually increases, the number of chain segments capable of moving freely within the CNT film and the CNT epoxy resin film also increases, leading to a decrease in the energy storage modulus of the material. Furthermore, the presence of additional voids within the CNT film exacerbates this phenomenon. As the temperature rises, the number of chain segments in the frozen or bound state decreases, resulting in reduced friction and a corresponding decline in the loss factor of the CNT. Conversely, in the CNT epoxy resin membrane, the epoxy resin occupies some of the voids, thereby creating relative friction between the CNT and the epoxy resin. As the temperature increases, the number of chain segments that can move freely also rises, which results in increased relative friction and an elevation in the loss factor.

In this experiment, carbon nanotubes (CNTs) devoid of surface functional groups were employed. These CNTs exhibit a six-membered carbon ring structure and demonstrate stable chemical properties, which impede their involvement in the cross-linking reaction with epoxy resin. Consequently, the carbon nanotubes do not establish covalent bonds with the epoxy molecules, or they form only a limited number of covalent bonds characterized by C-O linkages. As a result, the primary interfacial binding force is attributed to van der Waals interactions [27]. Within the CNT-epoxy resin composite, the epoxy resin infiltrates the internal structure of the CNTs, resulting in relative friction between the molecular segments of the CNTs and the epoxy resin. As the number of freely moving segments increases, so does the relative friction, which subsequently elevates the loss factor with rising temperature. Due to the differing thermal expansion coefficients of carbon nanotube fibers and epoxy resins, the radial thermal expansion deformation of the epoxy resin is significantly greater than that of the carbon nanotube fibers as ambient temperature increases. This disparity weakens the interfacial binding force, leading to a reduction in the interface strength of the fiber/resin [28], and then the number of freely moving segments results in heightened relative friction and an increase in the loss factor.

### 3.2. Effect of Frequency on Material Properties

The energy storage modulus $E^{\prime }$ and loss factor tanθ of pure CNT films, as well as CNT epoxy films at different frequencies, are compared and discussed below.

**Figure 8:**
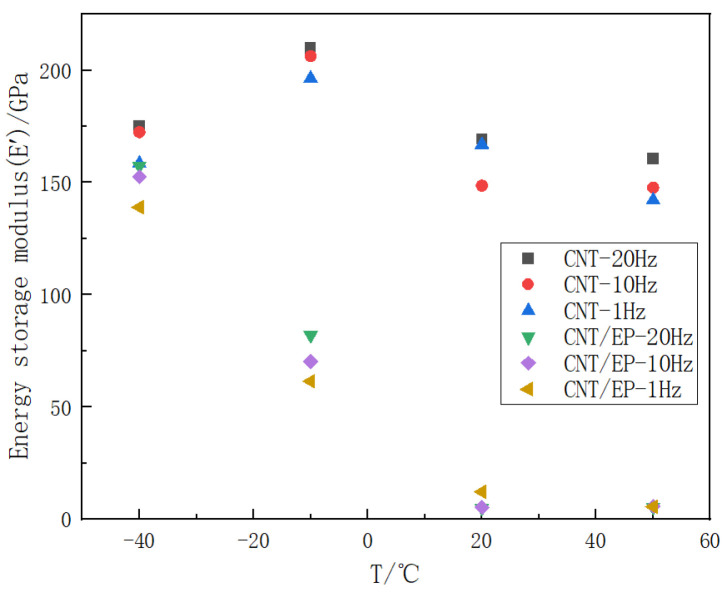
Temperature dependence of the energy storage modulus of CNT film and CNT epoxy resin film at different frequencies (1 Hz, 10 Hz, and 20 Hz).

**Figure 9:**
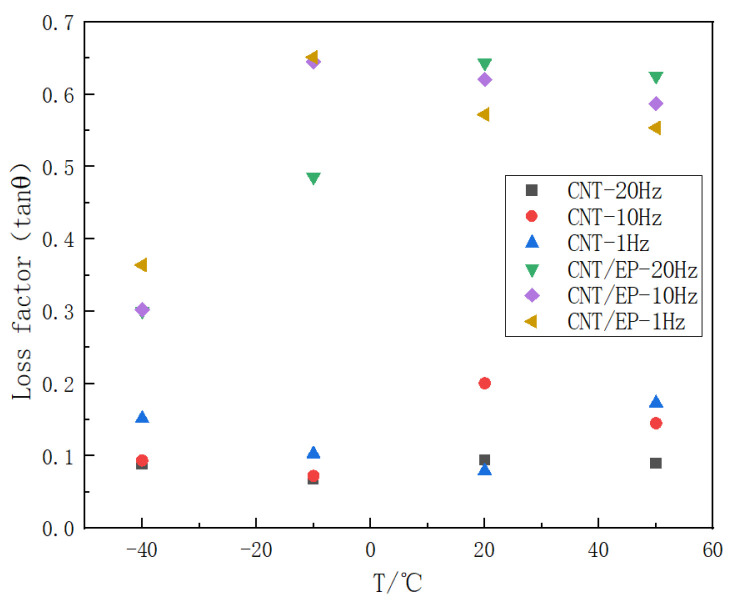
Temperature dependence of the loss factor of CNT film and CNT epoxy resin film at different frequencies (1 Hz, 10 Hz, and 20 Hz).

As illustrated in Figure 8 and Figure 9, the energy storage modulus and loss factor were obtained by comparing pure CNT films and CNT epoxy at different frequencies and ambient temperatures. At a constant ambient temperature, the energy storage modulus of both pure CNT films and CNT epoxy resin exhibits an increase with rising frequency, with an approximate increase ranging from 1% to 15%. Conversely, the loss factor demonstrates a reduction to different degrees with the increase in frequency.

As the frequency of the applied alternating load increases, the duration of this load becomes increasingly shorter than the relaxation time of the molecular chains and their segments within the material. As a result, the movement of the chain segments is unable to synchronize with the changing stress, which hinders the rearrangement of the molecular chains. This phenomenon leads to a decrease in friction among the molecular chains, an enhancement of the material’s rigidity, a reduction in energy loss, an increase in the energy storage modulus, and a decrease in the loss factor [29].

In comparison to other nano-reinforced composites, such as graphene oxide/nano cellulose composite films [30,31], the thermomechanical properties of carbon nanotube epoxy composites are significantly superior. This enhancement can be attributed to the exceptional characteristics of carbon nanotubes, despite the influence of temperature and frequency on their material properties. For instance, in the case of graphene foam nanomaterials [32], temperature and frequency exert minimal effects on the storage modulus and loss factor of the materials.

### 3.3. Effect of the Presence of Epoxy Resin on the Material Properties

**Figure 10:**
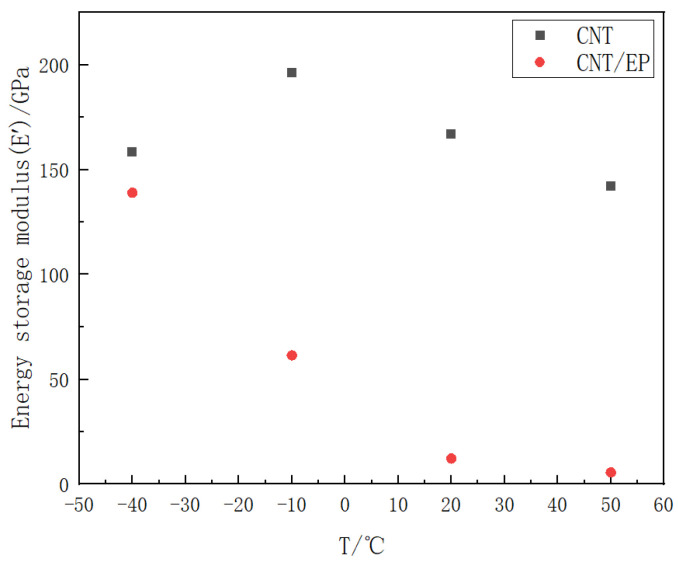
Comparison of the energy storage modulus under different temperature of CNT epoxy resin film and CNT film (−40 °C, −10 °C, 20 °C, and 50 °C).

As depicted in Figure 10, a comparative analysis of the energy storage modulus between pure carbon nanotube (CNT) films and CNT epoxy resin films, conducted at identical frequency and temperature conditions, indicates that the incorporation of epoxy resin leads to a decrease in the energy storage modulus of the carbon nanotube film. Specifically, the energy storage modulus of the pure CNT film is approximately 13 times greater than that of the composite material at 20 °C. The energy storage modulus serves as a measure of a viscoelastic material’s ability to store energy over one cycle under alternating stress; a higher energy storage modulus reflects an enhanced capacity for energy storage resulting from elastic deformation, as well as the material’s ability to return to its original shape when the stress is removed [29]. Consequently, the integration of epoxy resin into carbon nanotubes to form CNT epoxy composites occupies some of the voids within the carbon nanotubes, thereby reducing the overall material’s capacity to store energy from elastic deformation and hindering its ability to recover to its original configuration.

**Figure 11:**
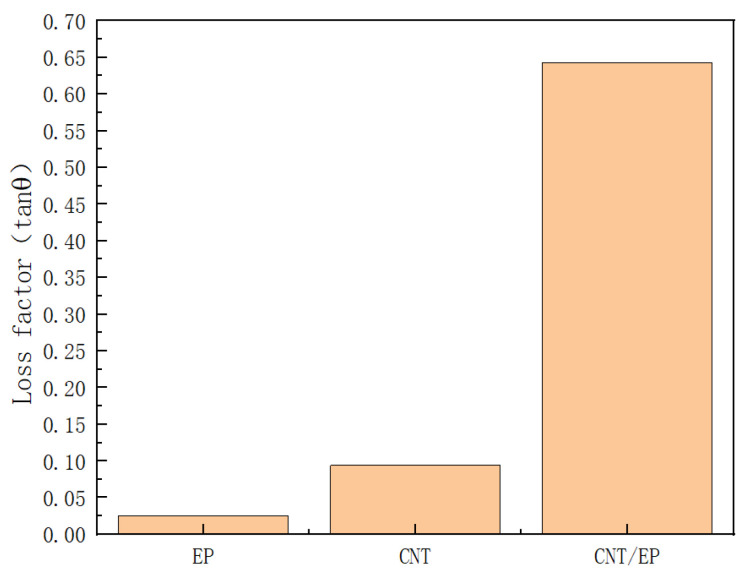
Comparison of the loss factors of epoxy resin, carbon nanotube, and CNT-epoxy resin.

The loss factor is defined as the ratio of energy dissipated per cycle to the maximum energy stored within a single cycle, thereby reflecting the dissipation of energy. A pronounced increase in the loss factor signifies a substantial amount of energy absorption, which serves a damping function [29]. The loss factor, commonly referred to as the damping factor, quantifies the damping performance of a material. A higher loss factor indicates superior damping performance, suggesting that the material exhibits greater viscosity and reduced elasticity. Existing research indicates that at 20 °C, the loss factor of pure epoxy resin is 0.025 [33]. As illustrated in Figure 11, a comparative analysis of the loss factors of pure carbon nanotubes, carbon nanotube-epoxy resin composites, and pure epoxy resin under identical conditions demonstrates that the incorporation of carbon nanotubes into the epoxy resin significantly enhances the loss factor. Specifically, the loss factor of the composite material at 20 °C is approximately 0.64, which is about 25 times greater than that of pure epoxy resin and 7 times greater than that of pure carbon nanotubes. This finding indicates that the addition of carbon nanotubes to the epoxy resin matrix improves the damping properties of the material.

The integration of epoxy resin into carbon nanotube films leads to a decrease in their elastic deformation capacity while simultaneously improving their damping characteristics. In contrast, pure carbon nanotube films demonstrate a greater capacity for elastic deformation when compared to carbon nanotube-epoxy resin composites. It is essential to highlight that carbon nanotube-epoxy resin composites differ from pure carbon nanotube films, which are noted for their advantageous damping properties.

## 4. Conclusions

In this study, CNT epoxy composite films were synthesized, and dynamic thermomechanical analysis (DMA) tensile tests were conducted. The analysis of the experimental data obtained elucidates the influence of epoxy on the mechanical properties of the films, in addition to the effects of temperature and frequency on these properties. The primary conclusions drawn from this research are as follows:

(1) The material properties of pure carbon nanotube films and carbon nanotube epoxy resin films are significantly affected by ambient temperature. At temperatures of −40 °C, −10 °C, 20 °C, and 50 °C, the energy storage modulus of pure carbon nanotubes reaches its peak value of approximately 196 GPa at −10 °C, where the material demonstrates optimal elastic deformation. As the temperature rises, the energy storage modulus of pure carbon nanotube films decreases by approximately 15%. In contrast, carbon nanotube epoxy resin films exhibit a marked reduction in energy storage modulus with increasing ambient temperatures. At lower temperatures, the energy storage modulus is roughly twice that observed at elevated temperatures, suggesting that the elastic deformation capacity of the composite material diminishes as ambient temperature increases.
(2) The material properties of pure carbon nanotube (CNT) films and CNT epoxy resin films are affected by variations in frequency. Specifically, at frequencies of 1 Hz, 10 Hz, and 20 Hz, an increase in frequency leads to a corresponding rise in the energy storage modulus of both the pure CNT film and the CNT epoxy resin film, with increases ranging from approximately 1% to 15%. In contrast, the loss factor exhibits a decrease to varying degrees as frequency increases.
(3) A comparative analysis of the material properties of pure carbon nanotube (CNT) film and CNT-epoxy composite film across varying temperatures indicates that the incorporation of epoxy resin has a significant impact on the film’s material properties. Specifically, this addition leads to a marked reduction in the energy storage modulus and an increase in the loss factor of the composite material. The energy storage modulus of pure CNT film is approximately 13 times greater than that of the composite material, whereas the loss factor of the composite is approximately 25 times higher than that of pure epoxy resin and 7 times higher than that of pure CNT film. The introduction of epoxy resin into the CNT film serves to fill the voids between the carbon nanotubes, thereby reducing the material’s capacity for elastic deformation during dynamic tensile testing. This filling process decreases the internal porosity of the material and introduces relative friction between the carbon nanotubes and the epoxy resin during tensile stretching, ultimately enhancing the damping performance of the material.
(4) The performance characteristics of carbon nanotubes (CNTs) and carbon nanotube epoxy resin composites at varying temperatures and frequencies provide a fundamental understanding of the operational mechanisms of CNT films. This understanding is essential when CNTs are utilized as reinforcing phase materials for interlayer toughening and the enhancement of impact resistance. Additionally, it establishes a theoretical framework for examining the performance variations in aircraft components that are predominantly composed of carbon nanotube epoxy resin composites under diverse temperature and operational conditions.

In this study, carbon nanotubes that lack surface functional groups are utilized, as the presence of such functional groups is likely to impact the interface between the materials and, consequently, influence their thermomechanical properties. Furthermore, considering the relative friction arising from the movement of chain segments, future research could investigate the frictional behavior between carbon nanotubes and epoxy resins, as well as the dynamic thermomechanical properties of carbon nanotube-epoxy resin composites that include surface functional groups.

## Data Availability

The original contributions presented in this study are included in the article. Further inquiries can be directed to the corresponding author.
